# Identification of Tympanic Border Cells as Slow-Cycling Cells in the Cochlea

**DOI:** 10.1371/journal.pone.0048544

**Published:** 2012-10-31

**Authors:** Mirei Taniguchi, Norio Yamamoto, Takayuki Nakagawa, Eriko Ogino, Juichi Ito

**Affiliations:** Department of Otolaryngology Head and Neck Surgery, Graduate School of Medicine, Kyoto University, Kyoto, Japan; Pomona College, United States of America

## Abstract

Mammalian cochlear sensory epithelial cells are believed to possess minimal regenerative potential because they halt proliferation during late stage of embryogenesis and never regenerate after birth. This means that sensorineural hearing loss caused by the death of cochlear sensory epithelial cells is a permanent condition. However, stem cells were recently identified in neonatal mice following dissociation of their inner ear organs. This suggests that regenerative therapy for sensorineural hearing loss may be possible. Unfortunately, dissociation distorts the microanatomy of the inner ear, making it difficult to determine the precise location of stem cells in unaltered specimens. To develop new therapeutic approaches based on sensory epithelial cell regeneration, the location of these stem cells must be elucidated. Stem cells normally proliferate at a slow rate in adult organs. In fact, so-called label-retaining cells, or slow-cycling cells, of the brain and skin are recognized as stem cells. In this study, using the exogenous proliferation marker, 5′-bromo-2′-deoxyuridine (BrdU) in combination with the endogenous proliferation marker Ki-67, we identified tympanic border cells. These cells, which are located beneath the basilar membrane *in vivo*, represent slow-cycling cells of the murine cochlea. Immunohistochemically, these cells stained positive for the immature cell marker Nestin. But it will be difficult to achieve regeneration of the cochlear function because these slow-cycling cells disappear in the mature murine cochlea.

## Introduction

**Figure 1:**
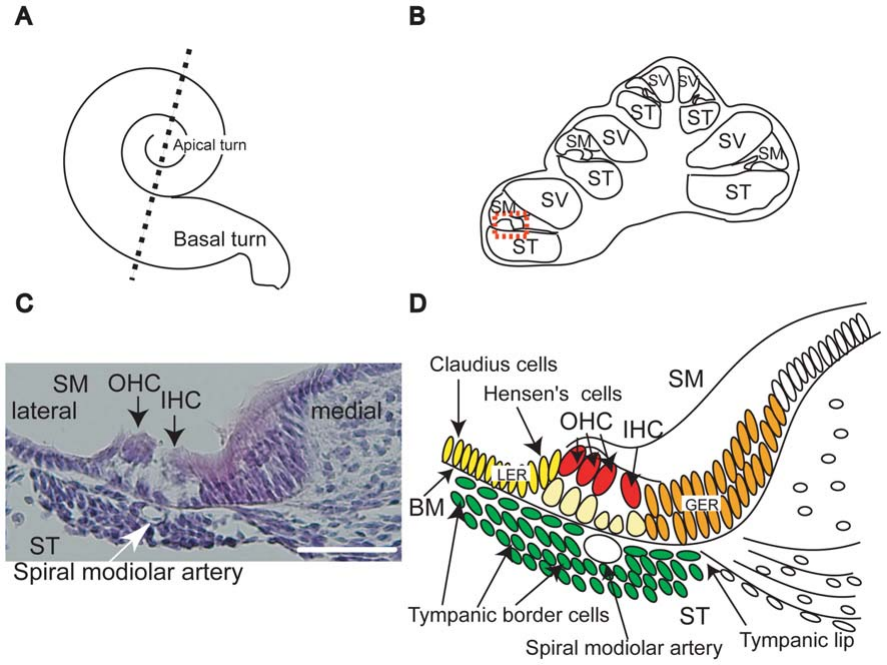
Anatomy of the cochlea. Illustrations of a whole cochlea and cochlear sections (A and B). B is an illustration of a section through dotted line in A. The cochlea exhibits spiral structure with several turns (A). The maturation of its sensory epithelia occurs in the basal to apical direction (A). The cochlea includes three spaces within its duct; scala tympani (ST), scala media (SM), and scala vestibuli (SV) (B). Hematoxylin and eosin stained section of an E18.5 cochlear basal turn (C) and an illustration (D) are presented. These are enlarged images of the orange dotted box in panel B. On the SM side of basilar membrane (BM in D) there are sensory epithelia composed of inner hair cells (IHC in C and D) and outer hair cells (OHC in C and D). The organ of Corti includes one row of IHCs and three rows of OHCs (red cells ind D). Supporting cells (SC) including Claudius and Hensen's cells exist lateral to (yellow cells in D) or under the hair cells (pale orange cells in D). Tympanic border cells (green cells in B) cover the ST side of the basilar membrane. The lateral and medial regions of the organ of Corti are referred to as lesser epithelial ridge (LER) (yellow cells) and greater epithelial ridge (GER) (orange cells), respectively. Below the organ of Corti, there is a spiral modiolar artery (C and D) surrounded by the tympanic border cells. Tympanic lip is the medial border of BM. The scale bar in C indicates 50 µm.

The mammalian inner ear consists of the cochlea and vestibules, which code for hearing and balance, respectively. Within the cochlea, the organ of Corti contains mechanosensory hair cells (HC) and surrounding supporting cells (SC) on the scala media (SM) side of the basilar membrane (BM) (Figure 1). Sensorineural hearing loss (SNHL) is predominantly caused by impairment of mechanosensory HCs within the cochlea. The regenerative potential of human cochlear HCs is limited to early developmental stages and doesn't occur in mature cochleae. In contrast, the adult vestibular organs exhibit a low level of HC genesis into adulthood.[1]–[4]

*Ex vivo* isolation of murine inner ear stem cells [5], [6] and progenitor cells [7] was recently achieved by dissociating inner ears of neonatal mice. Dissociated utricular maculae from mature animals and cochlear and vestibular organs from young animals gave rise to immature spheres that expressed various genetic markers of the developing and mature inner ears. p27^kip1^-positive cells, presumably SCs, that were isolated from dissociated neonatal cochleae incorporated BrdU. These p27^kip1^-positive cells transdifferentiated into Myosin7a-positive HCs. This suggests that mammalian cochlear SCs possess progenitor capabilities [8] similar to those seen in avian basilar papillae. Unfortunately, the location of the stem cell or progenitor cell populations is yet to be determined. Even in the experiments employing p27^kip1^ as a marker, it was difficult to determine which p27^kip1^-positive SCs constitute the progenitor cell population due to the use of dissociated inner ear cells and *ex vivo* analyses. Additionally, *ex vivo* studies cannot provide information regarding the physiological environment surrounding these cell types. We know from previous studies that the microenvironment is important in maintaining stem cell characteristics [9]. For this reason, if we are to succeed in the field of regenerative medicine, it is important for us to map stem cell locations and understand their surrounding environment.

Because stem cells are normally slow cycling [10], they have been identified *in vivo* through the use of the proliferation markers, tritium and BrdU. This technique has been successful in detecting stem cells in many organs, including corneal epithelia stem cells, hair follicular epithelial stem cells, prostate epithelial stem cells, hepatic stem cells, and mammary epithelial stem cells [11]–[15]. In this approach, a nucleotide analogue (such as BrdU) is injected into the subject, followed by an extended period (termed the long chase period) during which all rapidly dividing cells slowly lose labeling via dilution. The end result is that only slow-cycling cells and cell populations that differentiated immediately following the injection of the nucleotide analogue retain the label. By labeling with a second proliferating marker (such as Ki-67) after the long chase period, slow-cycling cells (label-retaining cells, or LRCs) can be identified *in vivo* (Table 1).

**Table 1:** Cellular types and patterns of proliferation markers expression.

	BrdU	Proliferation marker (Ki67)
Slow cycling cells	positive	positive
Rapidly dividing cells	negative (diluted out)	positive
Post-mitotic cells after BrdU injection	positive	negative
Non - dividing cells	negative	negative

Using this approach, we identified the location of slow-cycling cells in the murine inner ear.

## Results

To identify the location of slow-cycling cells in the murine inner ear, we sought to locate LRCs. To this end, we used BrdU and Ki-67 as proliferation markers. BrdU is a synthetic nucleoside and an analogue of thymidine; it becomes incorporated into the cell's DNA during the S phase of mitosis. Ki-67 is a nuclear proliferation antigen that is present during all active phases of the cell cycle (G_1_, S, G_2_, and mitosis). Thus, it is absent in resting cells (G_0_).

**Figure 2:**
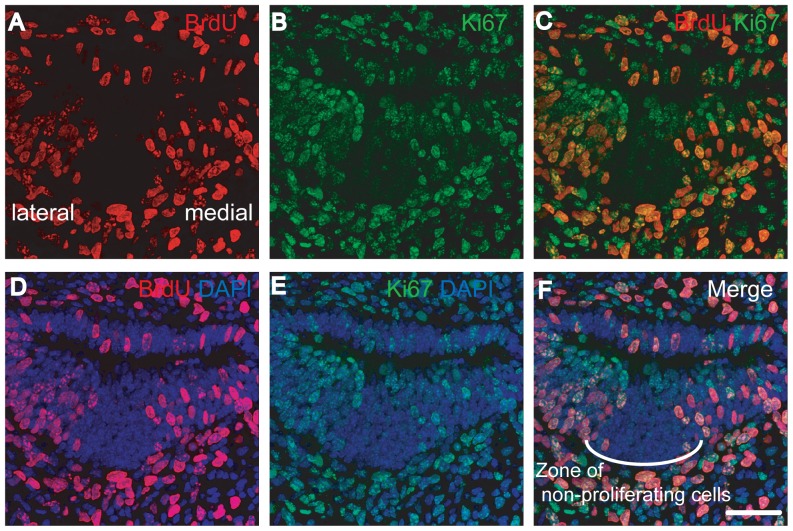
Section through an E13.5 murine cochlear duct one hour after BrdU injection. All panels are from one section. Double immunostaining was performed for BrdU (A, C, D, and F) and Ki-67 (B, C, E, and F) simultaneously, and nuclei were stained using DAPI (D, E, and F). C is a merged image of A and B. F is a merged image of D and E. We observed many BrdU and Ki-67-positive cells in the cochlear ducts (A and B) and almost all the BrdU-positive cells were Ki-67-positive (C). Both staining patterns clearly indicate a zone of non-proliferating cells (F) representing future sensory epithelial cells. The scale bar indicates 50 µm.

To validate the use of the two proliferation markers concurrently, we immunostained embryonic day 13.5 (E13.5) murine cochlear ducts with both anti-BrdU and anti-Ki-67 antibodies. This was completed one hour following the injection of BrdU. Our initial study demonstrated the presence of BrdU and Ki-67 in many cochlear ductal cells (Figure 2 A, B, D, and E). Co-localization of BrdU and Ki-67 was observed in nearly all of the BrdU-positive cells (Figure 2 C and F). Additionally, both staining patterns clearly showed a zone of non-proliferating cells (Figure 2 F). Previously, this zone of absent mitotic activity was shown to represent newly differentiated sensory epithelial cells [16]. Taken together, these results demonstrate that both BrdU and Ki-67 are reliable markers of proliferation.

To locate slow-cycling cells (or LRCs), we first injected BrdU one day before the cessation of sensory epithelial cell proliferation, on E13.5 [4]. At this time, we set a long chase period of five days.

After being collected on E18.5, the inner ear structures of the mouse embryos were double immunostained for BrdU and Ki-67. No morphological degeneration of the specimens was noted, indicating that BrdU did not affect inner ear differentiation. Following injection, BrdU was incorporated into rapidly dividing cells, differentiated cells that stopped proliferating immediately following BrdU injection (post-mitotic cells), and slow-cycling cells. BrdU was diluted out from rapidly dividing cells but was retained by post-mitotic and slow-cycling cells for five days following injection. At E18.5 post-mitotic cells were negative for Ki-67 due to the fact that post-mitotic cells cease proliferation immediately following BrdU injection at E13.5. Hence, only slow-cycling cells were positive for both BrdU and Ki-67 (Table 1).

**Figure 3:**
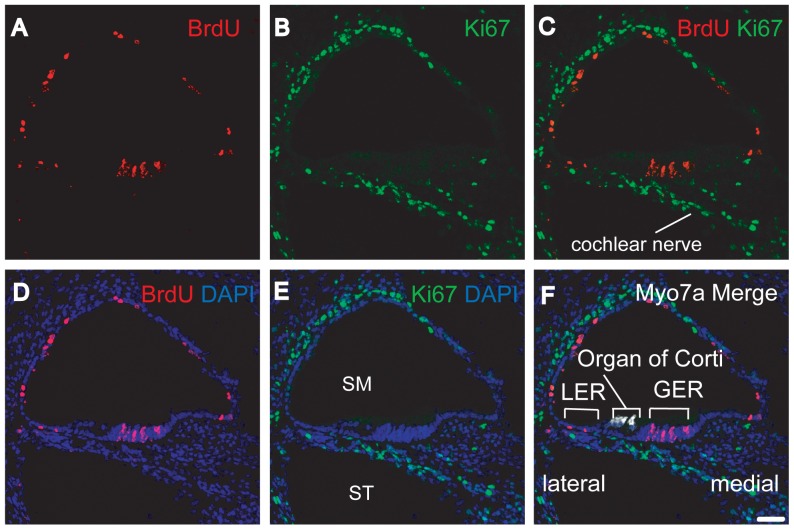
Section through an E18.5 cochlea five days after BrdU injection. All panels are from one section. This section is representative of the ten cochleae studied. Triple immunostaining was performed for BrdU (A, C, D, and F), Ki-67(B, C, E, and F), and Myosin 7a (Myo7a, hair cell marker) (F). Nuclei were stained using DAPI (D, E, and F). We observed BrdU-positive cells mainly lateral and medial to organs of Corti (A, C, D, and F) in the lesser epithelial ridge (LER) and greater epithelial ridge (GER) areas, respectively. In contrast, Ki-67-positive cells were found among the tympanic border cells lining the scala tympani (ST) side of basilar membrane, and among cochlear nerve fibers (B, C, E, and F). GER : greater epithelial ridge, LER : lesser epithelial ridge, SM : scala media, ST : scala tympani. The scale bar indicates 50 µm.

At E18.5, we found many BrdU-positive cells located lateral and medial to the organs of Corti (Figure 3 A, D and F). These regions are called lesser epithelial ridge (LER) and greater epithelial ridge (GER), respectively. The GER were reported to contain cells that can transdifferentiate into hair cells following over-expression of the atoh1 gene [17]. Meanwhile, cells within the LER have been shown to transdifferentiate following inhibition of Notch signaling [18]. We also found BrdU-positive cells in the stria vascularis, Reissner's membrane, spiral ligament, and spiral limbus. In contrast, tympanic border cells that line the scala tympani (ST) side of BM were Ki-67-positive (Figure 1 and Figure 3 B, E, and F). This is consistent with the findings of previous studies that reported the presence of proliferating tympanic border cells after birth [4], [19].

**Figure 4:**
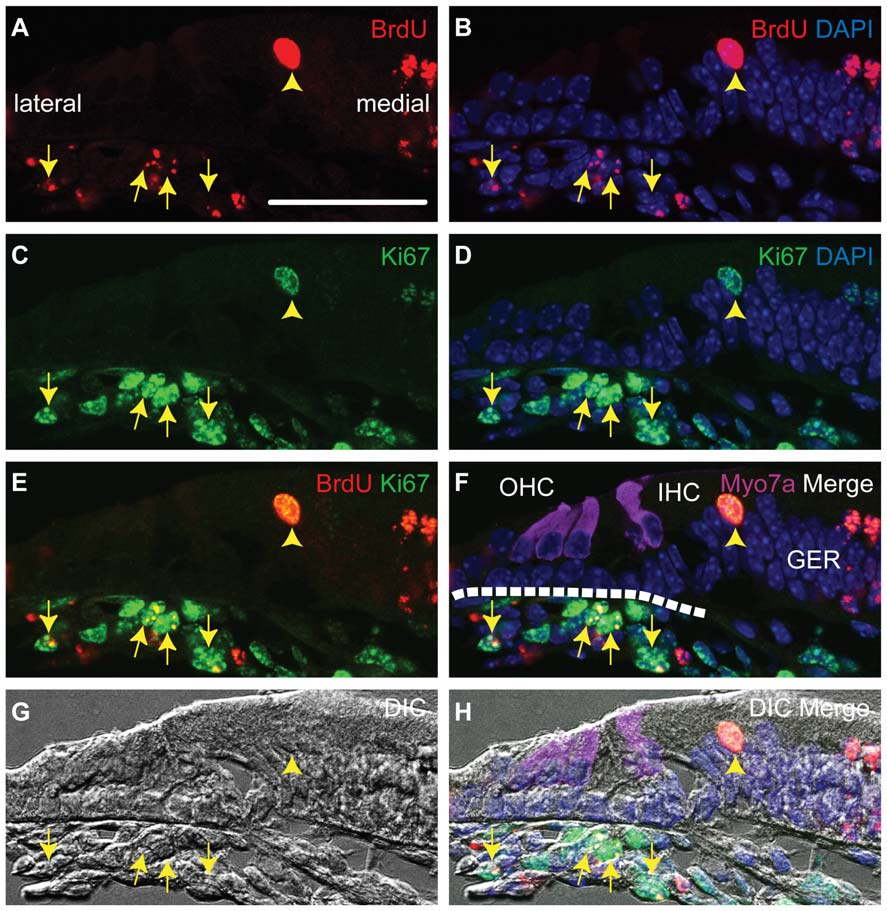
Slow-cycling tympanic border and GER cells (E18.5). All panels are from one section. This section is representative of the ten cochleae studied. Triple immunostaining was performed for BrdU (A, B, E, F, and H), Ki-67(C, D, E, F, and H) and Myosin7a (Myo7a, hair cell marker) (F and H). Nuclei were stained using DAPI (B, D, F, and H). The difference interference contrast image (G) and fluorescent signal detection image were taken at the same time and merged (H). We detected double-positive cells in the tympanic border cell populations (arrows) and the GER (arrowheads) in the E18.5 (BrdU administered at E13.5). GER: greater epithelial ridge, IHC: inner hair cell, OHC: outer hair cells, DIC: difference interference contrast image. The broken line in F indicates the basilar membrane. The scale bar indicates 50 µm.

We speculated that the numbers of cells showing double staining would be extremely small. Accordingly, we prepared four consecutive mid-modiolar sections from each of ten cochleae. In these forty cochlear sections, we found 63 double-positive (BrdU and Ki-67-positive) cells (LRCs) within tympanic border cells (Arrows in figure 4) and 2 double-positive cells (LRCs) within the GER (Arrowheads in figure 4). In all other locations within the cochleae, double-positive cells were absent. In conclusion, slow-cycling cells were predominantly seen within the tympanic border population between days E13.5 and E18.5.

**Figure 5:**
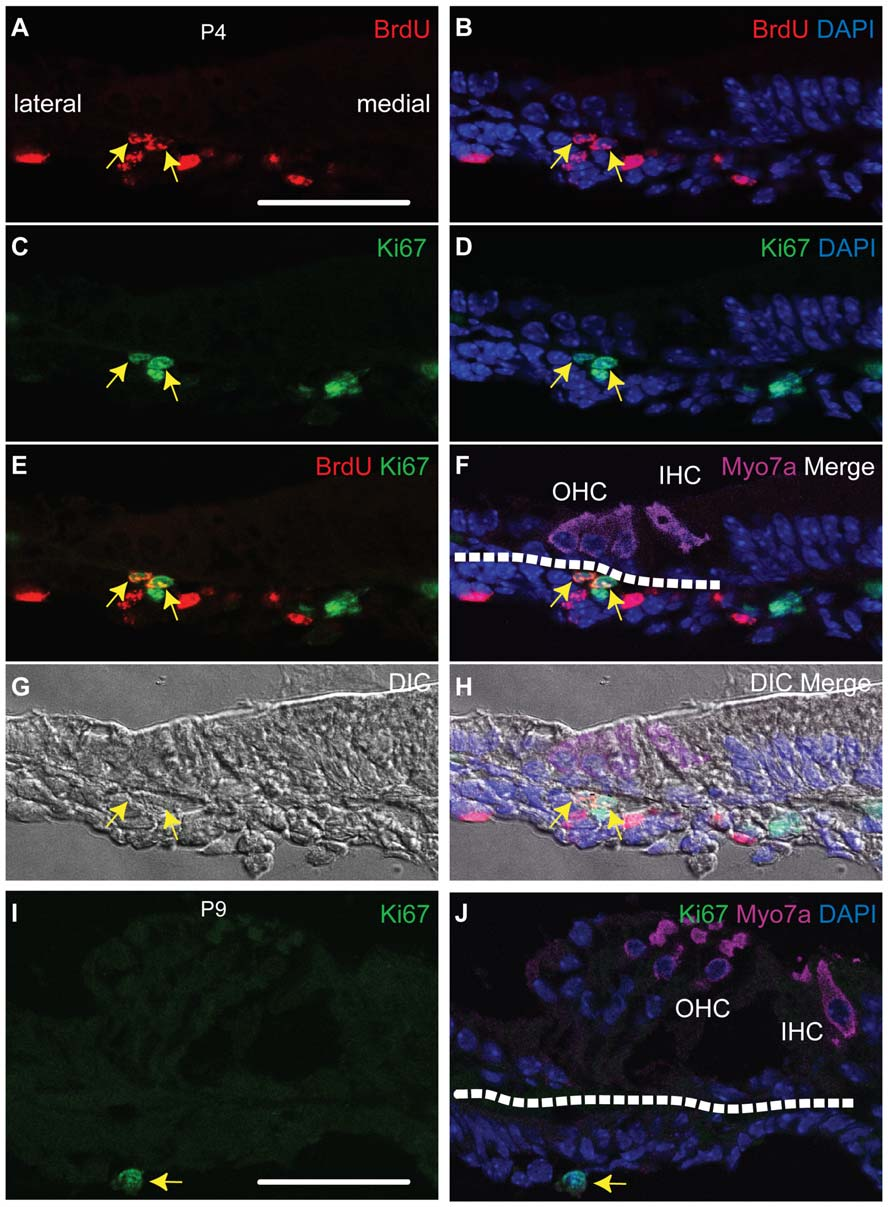
Postnatal slow-cycling cells of the tympanic border zone. Panels A-H (P4) are from one secion. Panels I and J (P9) are from a second section. These sections are representative of the cochleae studied. Triple (A-H) or double (I and J) immunostaining was performed for BrdU (A, B, E, F, and H), Ki-67(C, D, E, F, H, I, and J) and Myosin7a (Myo7a, hair cell marker) (F, H and J). Nuclei were stained using DAPI (B, D, F, H, and J). The difference interference contrast image (G) and fluorescent signal detection image were taken at the same time and merged (H). We observed double-positive cells within the tympanic border zone (arrows) in the P4 mouse cochlea (BrdU administered at E18.5) (A-H). We did not detect double-positive cells in the P9 mouse cochlea although there were few Ki-67-positive cells in the tympanic border zone (I and J). IHC: inner hair cell, OHC: outer hair cells DIC: difference interference contrast image. The broken lines in F and J indicate the basilar membrane. The scale bar indicate 50 µm.

Based on the above result, we sought to identify the location of slow-cycling cells during neonatal stages. To accomplish this, we next injected BrdU at E18.5 and collected cochlear tissues at P4. As in the experiment described above, 4 consecutive sections were obtained from a total of 10 cochleae. LRCs existed only within the tympanic border cell populations at P4 (Figure 5 A-H). The total number of LRCs during this time window was 23, compared to 63 during the embryonic stage (E13.5 through E18.5). When we performed the same experiments for the P4 through P9 time window, we did not find any double-positive cells (LRCs) (n = 5, data not shown). In fact, there were only scattered Ki-67-positive cells at the P9 time point (Figure 5 I and J). These results indicate that the existence of slow-cycling cells at early postnatal stages. They also suggest that either the cell cycle becomes slower or the numbers of LRCs are decrease as the cochlea matures.

**Figure 6:**
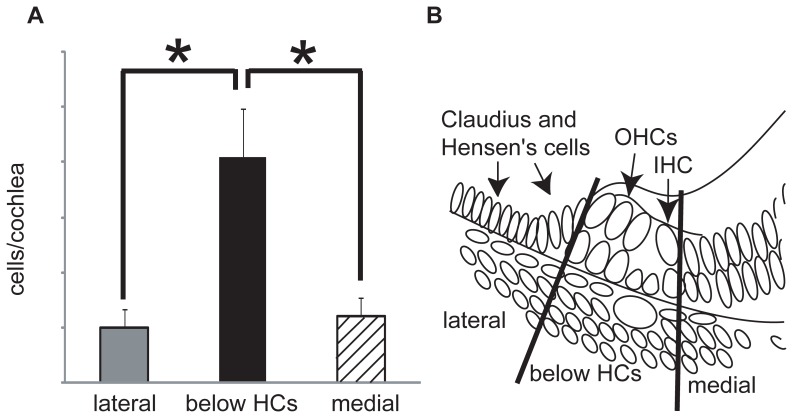
Localization of slow-cycling cells within tympanic border cell region. We counted the numbers of double-positive cells within three areas of the basilar membrane (A): from the region under the tympanic lip to under the medial side of the inner hair cells (medial region); from the region under the inner hair cells to the area under the outer hair cells (region below the HCs); and the region under the Hensen's and Claudius cells (lateral region) (B). Statistical analyses were performed using one-way analysis of variance (ANOVA) and multiple comparisons (Tukey-Kramer method). Roughly two-thirds of the observed slow-cycling cells (41/63) were located in the region below the hair cells where a cochlear spiral modiolar artery exists. The number of slow-cycling cells in the area below the HCs was significantly greater than was detected in other regions (A). * : p<0.01. Bars indicate the standard error of the mean. IHC: inner hair cell, OHC: outer hair cells.

To more precisely evaluate the location of double-positive cells within the tympanic border cell region, we subdivided the BM into three separate regions (Figure 6). Within each of these regions, we quantified the number of double-positive cells. The regions were as follows: from the area under the tympanic lip to those under the medial side of inner hair cells (medial region); from the area under the inner HCs to the area under the outer HCs (region below HCs); and finally the area under Hensen's and Claudius cells (lateral region) (Figure 1 and 6). A significantly greater number of double-positive cells (Figure 6, p<0.01, approximately two-thirds of all double-positive cells) were located in the region below the HCs where the cochlear spiral modiolar artery exists (Figure 1).

**Figure 7:**
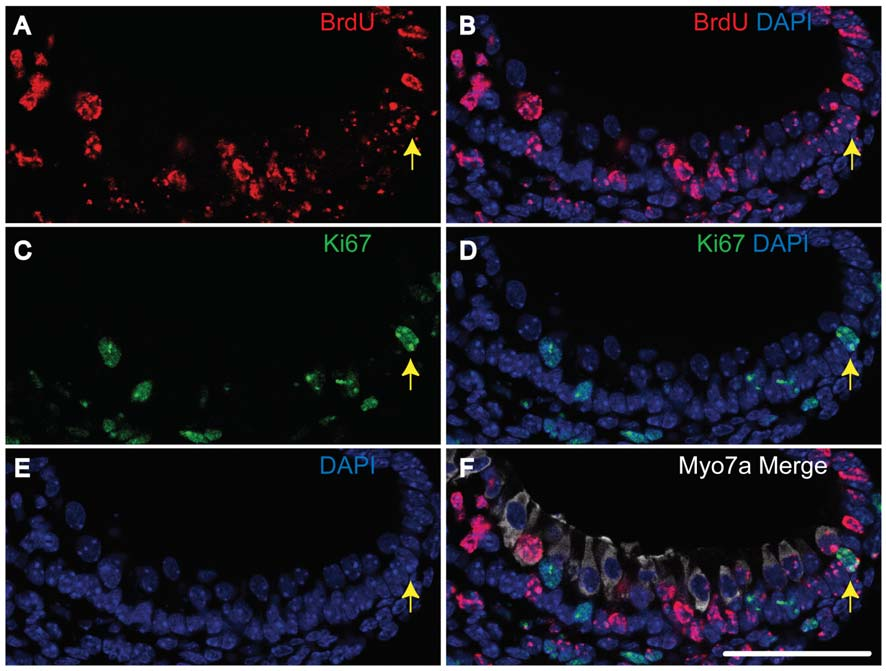
Slow-cycling cells in the supporting cell layer of the vestibular organ. All panels are from one section. Triple immunostaining was performed for BrdU (A, B, F), Ki-67 (C, D, F), and Myosin7a (Myo7a, hair cell marker) (F), and nuclei were stained using DAPI (B, D, E, F). We detected double-positive cells (arrows) in the supporting cell layer (below Myosin7a-positive hair cells) in the E18.5 mouse vestibular organ (BrdU administered at E13.5). The scale bar indicates 50 µm.

We then turned our attention to BrdU-Ki-67 double-positive cells within vestibular system. It has been reported that even in mammals, such organs exhibit a postnatal regenerative capacity [1], [2]. Additionally, the vestibular organs retain a stem cell population later in development [6]. To quantify the number of double-positive cells, we used saccular maculae. Within 16 saccular maculae-sections, we found 38 double-positive cells in the supporting cell layer. These 16 sections from E18.5 animals represented four consecutive sections each from four saccules (Figure 7).

**Figure 8:**
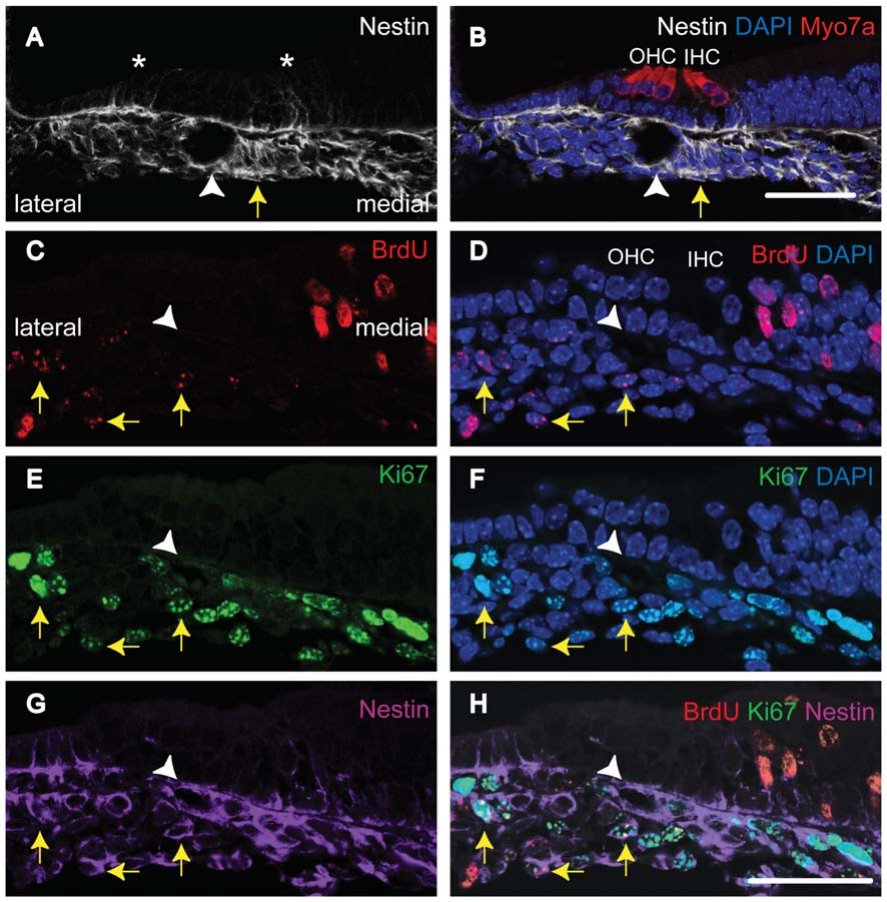
Nestin-positive tympanic border cells (E18.5). Panels A and B are from the same section. Panels C-H are from a second section. Double immunostaining (A and B) for Nestin and Myosin7a (Myo7a, hair cell marker), or triple immunostaining (C-H) for BrdU (C, D, and H), Ki-67 (E, F, and H), and Nestin (G and H) was performed. Nuclei were stained using DAPI (B, D, and F). Tympanic border cells positive for Nestin (arrows in A and B). Most of the BrdU-Ki-67-double positive tympanic border cells are also positive for Nestin (arrows in C-H). The white arrowheads indicate a cochlear spiral modiolar artery. Asterisks in A and B indicate Nestin-positive cells in GER and LER. The scale bar indicates 50 µm.

**Figure 9:**
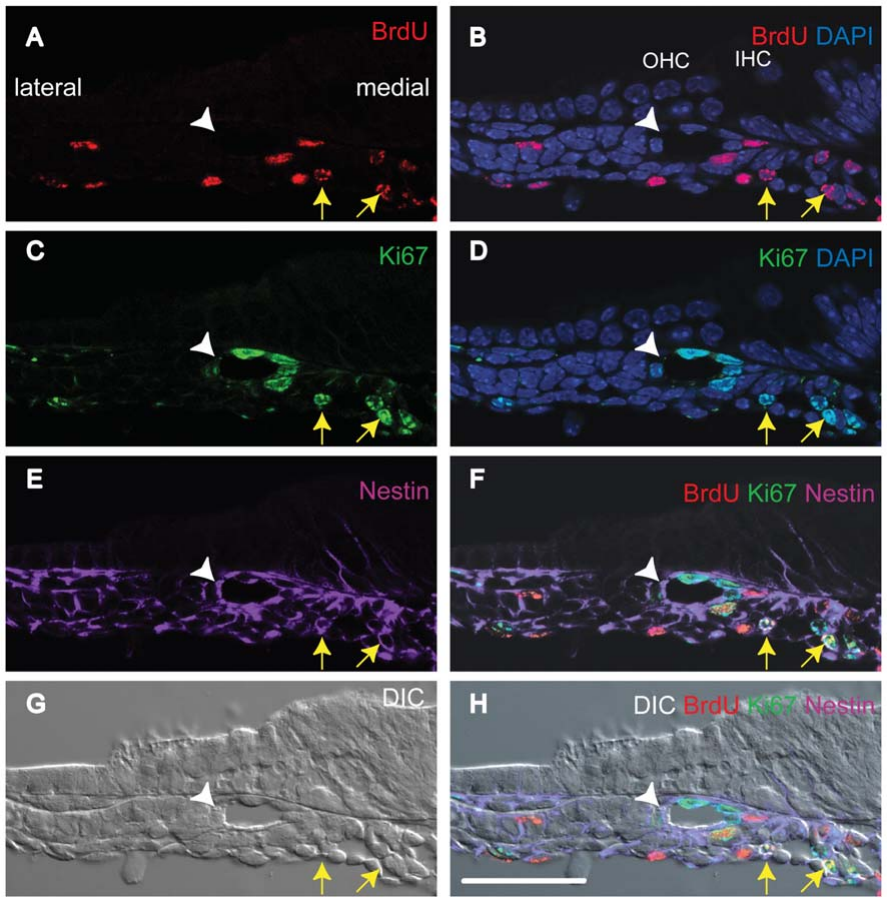
Nestin-positive tympanic border cells (P4). All panels are from one section. This section is a representative result of the ten cochleae studied. Triple immunostaining was performed for BrdU (A, B, F, and H), Ki-67(C, D, F, and H) and Nestin (E, F and H). Nuclei were stained using DAPI (B, D, F, and H). The difference interference contrast image (G) and fluorescent signal detection image were taken at the same time and merged (H). We observed triple-positive cells in the tympanic border zone (arrows) in the P4 mouse cochlea (BrdU administered at E18.5). IHC: inner hair cell, OHC: outer hair cells, DIC: difference interference contrast image. The white arrowheads indicate a cochlear spiral modiolar artery. The scale bar indicates 50 µm.

To characterize the tympanic border cells, we performed immunohistochemistry to detect Nestin (Figure 8 and 9), E-Cadherin (data not shown), and vimentin (data not shown) at E18.5 and P4. Nestin is a class 4 intermediate filament protein originally identified in neuroepithelial stem cells [20]. E-Cadherin and vimentin are markers for epithelial and mesenchymal cells, respectively. We found that some tympanic border cells were Nestin positive cells (Figure 8 A and B). However, neither E-cadherin nor vimentin was detected in tympanic border cells (data not shown). These results reflect the immature nature of tympanic border cells. To confirm that Nestin-positive cells are indeed slow-cycling cells, we performed triple immunohistochemistry for Nestin, Ki-67, and BrdU at E18.5 (Figure 8 C-H) or P4 (Figure 9). Most of the BrdU-Ki-67 double-positive cells (LRCs) within the tympanic border cell populations were, in fact, positive for Nestin at both E18.5 and P4.

**Figure 10:**
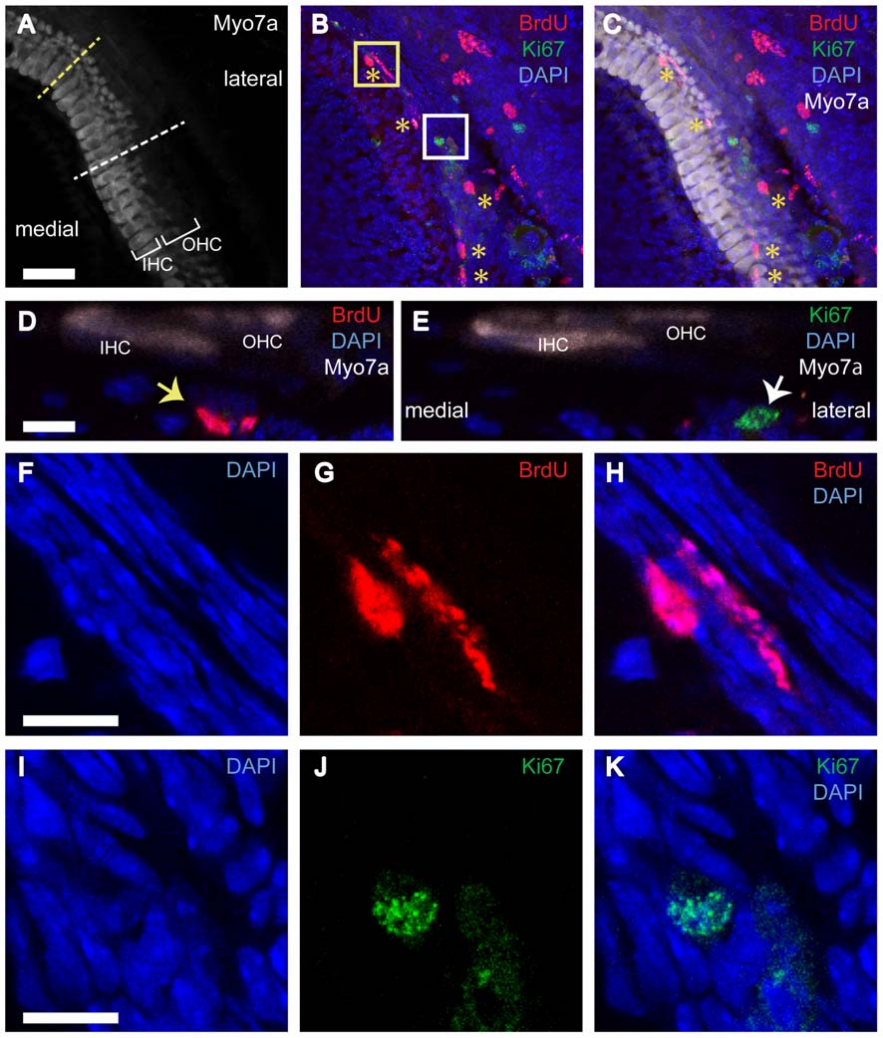
Proliferation status of tympanic border cells in the mature cochleae. Triple immunostaining was performed on the whole mount cochlea of four-week-old mice for Myosin7a (Myo7a, hair cell marker) (A, C, D and E), BrdU (B-D, G and H), and Ki-67(B, C, E, J, and K). Nuclei were stained using DAPI (B-E, G, H, I, and K). Stacked images of the whole mount cochlea (A-C) showed that many BrdU-positive cells (asterisks in B and C, BrdU administered from P3 to P5) and one Ki-67-positive cell (white boxed cell in B) were located around hair cells. Re-sliced images of A-C (D and E, re-sliced at yellow and white dotted lines in A, respectively) showed that these BrdU-positive cell (yellow arrow in D) and Ki-67-positive cell (white arrow in E) were located at the level of tympanic border cells. Optical sections of the whole mount cochlea showed that BrdU- and Ki67- signals were those from nuclei (F-K). F-H and I-K were optical sections at the regions of yellow and white box in B, respectively. IHC: inner hair cells, OHC: outer hair cells. The scale bar in A indicates 30 µm. The other scale bars in D, F, and I indicate 5 µm.

Finally, we examined cochlear tissue from adult mice in order to determine whether Ki-67-expressing tympanic border cells exist in the mature cochlea, and whether they retain labeling of BrdU. We injected BrdU once a day during P3-5, and examined specimens fixed at P28. Because we expected very few Ki-67-positve cells, specimens were processed as whole-mounts, in order to view the entire organ of Corti. We found many BrdU-positive tympanic border cells (Figure 10 B, C, D, G, and H), indicating at least some of the cells that incorporated BrdU at P3-5 did not migrate away but remained near the basilar membrane. We also found three Ki-67-positive cells among tympanic border cells (Figure 10 B, C, E, J, and K) from 6 cochleae. There were no Ki67 positive cells at other cell populations, but we did not observe any BrdU-Ki-67-double positive cells.

## Discussion

Better characterization of inner ear stem cells may lead to the development of treatment options for SNHL. Several groups have reported the existence of stem cells within the cochleae thus far [5]–[7]. Unfortunately, such reports have relied on dissociation of the inner ears, leading to distorted microanatomy. Thus, the location of the continuously proliferating cell populations was undetermined prior to the present study. In this study, we adopted a strategy to locate and identify so-called LRCs. Previously, stem cells were identified in corneal epithelia [11], hair follicular epithelia [12], prostate epithelia [14], mammary gland epithelia [15], and liver [13] using this method. These label-retaining slow-cycling cells have been shown to have high proliferative potential *in vitro* [21]–[24], which is another important characteristic of stem cells.

Using BrdU and Ki-67, we were able to locate slow-cycling cells within the tympanic border cell population of the cochlea at least until the neonatal stage.

Tympanic border cells have been shown to proliferate following injury to the inner ears in chickens [25], gerbils [26], rats, and guinea pigs [27]. Originally, tympanic border cells were defined as those cells lining the ST side of the basilar membrane [28]. However, the definitive characteristics of this cell population were previously unknown. In chickens, these cells continue to proliferate through the normal life cycle [25]. When acoustic injuries are applied to the inner ears, the proliferation of tympanic border cells increases markedly [25]. This suggests tympanic border cells respond to acoustic injuries to the inner ears. Even in mammals that do not show any regeneration capacity in the cochlea, tympanic border cells have been observed to proliferate after acoustic or drug-induced injuries to the cochlea [26], [27]. Other regions of the mammalian cochlea, including the stria vascularis, spiral ligament, and acoustic nerve fibers, exhibit proliferating cells following cochlear injury [26], [27]. In the current study, we were unable to locate significant numbers of slow-cycling cells in these regions.

Within the cochlea, we located several Nestin-positive cell populations, including a Nestin-positive tympanic border cell population (Figure 8 C-H and figure 9). Nestin is a marker of immature proliferating cells of many different types including neural tissues [20] and muscles.

In the cochlea, a GFP signal under control of the Nestin promoter was detected in a previous study in other cochlear cell populations, including SCs and GER cells [29]. Both the GER cells and tympanic border cell populations contained slow-cycling cells. However, the numbers of these cells within GER were considerably smaller compared to the tympanic border cells. As previously noted, GER region is a source of ectopic hair cells when induced by over-expression of atoh1 [17], [30]. Given the ability to generate hair cells, the GER region is yet another prospective region of stem/progenitor cells. We did not find any slow-cycling cells among the SCs of the sensory epithelium.

In addition to Nestin, Axin2 has been found in postnatal tympanic border cells [31]. As a target of Wnt signaling, Axin2 regulates progenitor cell growth and maturation in several tissue types, including prostate tissue [32] and the late primitive streak [33]. Tympanic border cells were negative for the epithelial marker, E-cadherin, and a mesenchymal marker, vimentin, demonstrating the immature nature of these cells.

Roughly 66% of the slow-cycling cells within the tympanic border zone were located near both a vascular structure (i.e., a cochlear spiral modiolar artery) and the basement membrane of the BM. The surrounding vasculature of the stem-cells has been shown to supply them with growth and maintenance signaling. [34], [35]. Blood vessel proximity also allows stem cells to sense distant changes and react accordingly. Additionally, the basement membranes, one of the components of the cochlear BM, provide scaffolding for the migration of stem cells as shown in the *Drosophila* intestine [36], [37]. In the same way, the microenvironment surrounding the most slow-cycling cells seems to have a significant role for them to keep proliferating, and differentiate into whatever mature cells. We cannot conclude which cells the tympanic border zone differentiates into based on the results of the present study.

The number of LRCs decreases as the cochlea develops and matures, raises two possibilities that these cell populations die off as maturation goes on, or that the rate of cell cycling gets much slower with the onset of cochlear maturation. To distinguish between these two possibilities, we employed experimental designs including longer BrdU labeling time and extended chase periods (Figure 10). We did not detect any BrdU-Ki-67-positive cells in tympanic border cells, consistent with the previous studies showing that the regenerative ability within the cochlea is extremely limited.

In conclusion, we found slow-cycling cells in the embryonic and neonatal murine cochlea within the tympanic border cell populations. Further studies are needed to characterize these tympanic border cells.

## Materials and Methods

### Animals

We purchased time-mated pregnant ICR mice from Japan SLC Inc. (Hamamatsu, Japan).

The experimental protocols were performed in accordance with the National Institutes of Health Guidelines for the Care and Use of Laboratory Animals and approved by the Animal Research Committee of Kyoto University Graduate School of Medicine (No. 11179).

### Incorporation of BrdU

For the embryonic and neonatal mice study, two 50 µg/g BrdU (BD Pharmingen) injection were administered intraperitoneally. These injections were spaced one hour apart. The ages of the experimental animals at the time of injection were E13.5, E18.5, and P4. The animals were then sacrificed five days post-injection and their inner ear structures were collected and analyzed for BrdU incorporation. At least five animals (ten cochleae) were analyzed for each experimental condition.

For the mature mice study, 50 µg/g BrdU was injected once a day from P3 to P5. The animals were then sacrificed at the age of four weeks and their inner ear structures were collected and analyzed for BrdU incorporation. Six cochleae were analyzed.

### Histological preparations

Mice were deeply anesthetized with carbon dioxide gas and euthanized. Inner ear structures were collected from their embryos or themselves. To obtain cryosections, the tissue was fixed in 4% paraformaldehyde (PFA) in phosphate buffered saline (PBS) for 4 hours at 4°C and dehydrated through graded concentrations of sucrose in PBS. The tissues from P9 were decalcified after fixation with 0.1 M EDTA (ethylenediaminetetraacetic acid) for 3 days. At the time of sectioning, the specimens were embedded in frozen OCT compound (Sakura Finetek Japan, Tokyo, Japan) and sectioned at a thickness of 10 µm.

For whole-mount preparations, embryonic or postnatal cochlear epithelia and vestibular epithelia were fixed at room temperature using 4% PFA in PBS for 15 minutes. For adult whole-mount preparations, cochlear epithelia was fixed for four hours in 4% PFA in PBS at 4°C followed by decalcification with 0.1 M EDTA for 5 days. After removing lateral walls and Reissner's membrane, the samples were immunostained.

### Immunohistochemistry

Antigen retrieval of BrdU was performed by incubating specimens at 98°C in citrate buffer (PH6.0) for 15 minutes. They were then held at room temperature for 20 minutes.

The primary antibodies used in this study were as follows: rat monoclonal anti-BrdU antibody (1∶20, Oxford Biotechnology), mouse monoclonal anti-Ki-67 antibody (1∶100, Novocastra), rabbit monoclonal anti-Ki-67 antibody (1∶100, Thermo Scientific), rabbit polyclonal anti-myosin7a antibodies (1∶500, Proteus), mouse monoclonal anti-nestin antibody (1∶500, BD Pharmingen). The secondary antibodies used in this study were Alexa-488-conjugated anti-mouse/rabbit IgG antibodies (1∶500, Molecular Probes), Alexa-555- or 568-conjugated anti-rat/rabbit/mouse IgG antibody (1∶500, Molecular Probes), and, Alexa-633-conjugated anti-rabbit IgG antibody (1∶500, Molecular Probes).

PBS containing 2 mg/mL of DAPI (Invitrogen) was used to stain nuclei. All images including difference interference contrast images were obtained using a confocal laser-scanning microscope (TCS SPE, Leica Microsystems Inc., Wetzlar, Germany). Re-slice images of whole-mount samples were obtained using ImageJ software (http://rsbweb.nih.gov/ij/index.html).

### Cell counts and statistical analysis

We prepared four consecutive mid-modiolar sections from each of ten cochleae (E18, and P4) and five cochleae (P9) and counted BrdU-Ki-67-double positive cells. To determine which tympanic border cell populations contained the greatest numbers of slow-cycling cells, we divided the tympanic border cells into three regions based on their location within the basilar membrane. After performing a one-way repeated-measures ANOVA, we performed multiple comparisons using the Tukey-Kramer method. We set the level of significance at p = 0.01.
